# Detecting Epidemic Malaria, Uganda

**DOI:** 10.3201/eid1305.061410

**Published:** 2007-05

**Authors:** Jonathan Cox, Tarekegn Abeku, James Beard, James Turyeimuka, Enoch Tumwesigye, Michael Okia, John Rwakimari

**Affiliations:** * London School of Hygiene and Tropical Medicine, London, UK; † Kabale District Health Management Team, Kabale, Uganda; ‡ Ministry of Health, Kampala, Uganda

**Keywords:** Malaria, epidemic, surveillance, early detection, Uganda, letter

**To the Editor:** In the field of malaria epidemic early warning, there exists an unfortunate but frequently accurate perception that health systems in many affected countries learn of epidemics by way of the popular press rather than through formal disease surveillance systems. Malaria epidemics are often easily recognized (albeit too late) by laypersons (*1*), but most routine disease surveillance systems lack the ability to provide accurate, timely indications of aberrations in case numbers. The World Health Organization (WHO) has set specific targets for early detection and control of malaria epidemics as part of a wider strategy to cut the global extent of malaria in half by 2010 (*2*). We describe experiences during a recent epidemic in southwest Uganda and examine the performance of a pilot early detection system.

In 2002, the Ugandan Ministry of Health began developing and piloting a new district-level malaria monitoring system in Kabale and Rukungiri (*3*). Located in Uganda’s southwestern highlands, these districts have experienced several serious malaria epidemics in recent years, most notably during the El Niño year of 1998 (*4*,*5*). In this new system, data generated from representative health facilities are collated, entered on computer, and analyzed by district teams on a weekly basis. Incoming data on clinical malaria are compared with a baseline of historical illness data from which the effects of long-term temporal trends have been removed, and an objective anomaly measure, or “standardized departure,” is used to provide a simple, intuitive index of deviation from expected weekly levels of incidence (*3*). Electronic reports are disseminated by email to the National Malaria Control Programme (NMCP) and others, including WHO and the United Nations Children’s Fund.

The monitoring system detected 2 malaria outbreaks in Kabale, 1 each in 2005 and 2006. During the most recent outbreak, the first warnings of abnormally high malaria incidence were communicated from the district team to the NMCP on June 5, 1 month before reports of the outbreak appeared in the press and >2 weeks before case numbers began to peak (Figure, panel A). In the 6 weeks from May 29 to July 9, Kabale’s 5 sentinel sites recorded 4,637 clinical malaria cases, 159% more than expected for this period. Although the sentinel network consists of health centers with limited inpatient facilities, available data on admissions showed a similar temporal pattern, with 616 patients admitted during the same 6-week period, 188% more than expected.

Although the ability of the system to generate timely epidemic warnings is encouraging, data from 1 sentinel site highlighted a potential limitation of using routine data from clinical diagnoses of malaria as a basis for epidemic detection. As elsewhere, routine outpatient data for Bufundi Health Centre suggested the occurrence of a malaria outbreak starting in early June and peaking in early July of 2006 (Figure, panel B). The slight delay in the onset of the outbreak at this site was plausible, given its high elevation (2,200 m) and geographic remoteness. Data for patients with parasitologically confirmed malaria, available through an ongoing malaria transmission study at Bufundi Health Centre (*3*), showed a different temporal pattern of incidence, however. As part of this study, all samples from malaria case-patients identified through clinical diagnosis were subject to a Paracheck-Pf immunoassay test (Orchid Biomedical Systems, Verna, Goa, India). Results indicated that, at the peak of the apparent malaria outbreak, the percentage of samples from clinically diagnosed cases that produced a positive diagnostic test was as low as 4% (Figure, panel B). These results are unlikely to reflect poor diagnostic performance of the testing (*6*); febrile illness other than malaria was likely the cause of the outbreak.

Recent experiences in Kabale also highlight the potentially unwieldy nature of indoor residual spraying campaigns in the absence of spatial targeting. In Kabale, a district-wide spraying campaign supported by the US President’s Malaria Initiative (*7*) was planned for the 2006 transmission season. However, shortages of trained personnel and other institutional delays meant that spraying could not begin until the third week of June, by which time the epidemic had peaked (and densities of vector mosquitoes had presumably begun to fall). By July 17, <50% of the targeted structures had been sprayed. In the future, careful targeting of spraying to areas of highest epidemic risk might lead to more timely completion of spraying activities. It might also be beneficial to create special spray teams that can respond quickly to specific alerts.

Recent experiences in Kabale have underlined the potential value of simple monitoring tools for early detection of epidemics but have also shown potential barriers to effective epidemic control. Our findings highlight the need to build systems that improve routine collection of parasitologically confirmed malaria data and allow rapid investigation of anomalies in incoming clinical data. It is equally important to develop procedures that translate early warning information into timely decisions concerning which epidemic control measures to use and how best to target them (*8*). Without these procedures, the value of early detection will be seriously undermined.

**Figure:**
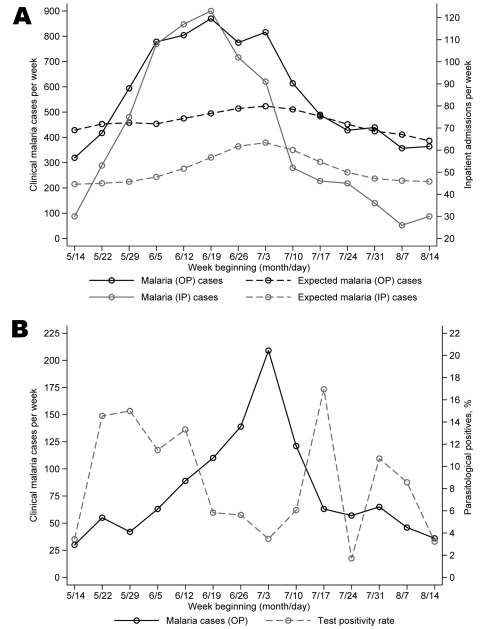
(A) Weekly observed and expected numbers of outpatient (OP) and inpatient (IP) cases of clinically diagnosed malaria from to 5 sentinel health centers in Kabale district, southwestern Uganda, May–July 2006. B) Weekly numbers of clinically diagnosed malaria cases and the proportion of cases subsequently testing positive for *Plasmodium falciparum* infection by rapid diagnostic test at Bufundi, Kabale district, southwestern Uganda, May–July 2006.
